# Dietary patterns of early childhood and maternal socioeconomic status in a unique prospective sample from a randomized controlled trial of Prenatal DHA Supplementation

**DOI:** 10.1186/s12887-016-0729-0

**Published:** 2016-11-25

**Authors:** Brandon H. Hidaka, Elizabeth H. Kerling, Jocelynn M. Thodosoff, Debra K. Sullivan, John Colombo, Susan E. Carlson

**Affiliations:** 1 Department of Dietetics and Nutrition, University of Kansas Medical Center, MS 4013, 3901 Rainbow Blvd., Kansas City, KS 66160 USA; 2 Department of Psychology, University of Kansas, 1415 Jayhawk Blvd., Lawrence, KS 66045 USA

**Keywords:** Dietary pattern, Empirically derived, Multivariate, Early childhood, Children, Socioeconomic status

## Abstract

**Background:**

Dietary habits established in early childhood and maternal socioeconomic status (SES) are important, complex, interrelated factors that influence a child’s growth and development. The aim of this study was to define the major dietary patterns in a cohort of young US children, construct a maternal SES index, and evaluate their associations.

**Methods:**

The diets of 190 children from a randomized, controlled trial of prenatal supplementation of docosahexaenoic acid (DHA) were recorded at 6-mo intervals from 2-4.5 years by 24-h dietary recall. Hierarchical cluster analysis of age-adjusted, average daily intake of 24 food and beverage groups was used to categorize diet. Unrotated factor analysis generated an SES score from maternal race, ethnicity, age, education, and neighborhood income.

**Results:**

We identified two major dietary patterns: “Prudent” and “Western.” The 85 (45%) children with a Prudent diet consumed more whole grains, fruit, yogurt and low-fat milk, green and non-starchy vegetables, and nuts and seeds. Conversely, those with a Western diet had greater intake of red meat, discretionary fat and condiments, sweet beverages, refined grains, French fries and potato chips, eggs, starchy vegetables, processed meats, chicken and seafood, and whole-fat milk. Compared to a Western diet, a Prudent diet was associated with one standard deviation higher maternal SES (95% CI: 0.80 to 1.30).

**Conclusions:**

We found two major dietary patterns of young US children and defined a single, continuous axis of maternal SES that differed strongly between groups. This is an important first step to investigate how child diet, SES, and prenatal DHA supplementation interact to influence health outcomes.

**Trial registration:**

NCT00266825. Prospectively registered on December 15, 2005

**Electronic supplementary material:**

The online version of this article (doi:10.1186/s12887-016-0729-0) contains supplementary material, which is available to authorized users.

## Background

Childhood has been widely studied as a critical period for establishing dietary habits that carry short and long term health consequences. However, the complexity of dietary intake data can cloud the connection between intake and health outcomes. Many European colleagues have begun to use multivariate statistical methods to consolidate large amounts of information about food and drink intake into dietary patterns that have been linked to pediatric health outcomes [1–6]. Heretofore, there are no examples of the use of these data-driven approaches to characterize dietary patterns among US children.

Socioeconomic status (SES) confounds efforts to isolate how childhood dietary habits relate to health outcomes. SES is a multidimensional construct that conveys relative wealth, power, and prestige, and it is influenced by race, ethnicity, parental education, occupation, and income. It is also a strong determinant of health [7] and has been repeatedly linked to diet quality [8–14]. Importantly, the association between SES and a health outcome can differ depending on how SES is measured [15]. Furthermore, the overall effect of SES cannot be accurately estimated when the highly collinear variables that determine SES are all added as covariates in a statistical model.

There is steadily accumulating evidence that the intrauterine environment has long-lasting effects on child growth and development (see [16] for just one recent review). We are extensively phenotyping a cohort of children from a randomized controlled trial of high dose prenatal docosahexaenoic acid (DHA) supplementation [17]. Given that the relationship between long-chain n-3 polyunsaturated fatty acids (n-3 LCPUFA) and a health outcome can hinge on environmental and genetic circumstance [18–20], it is possible that the effect of prenatal supplementation differs depending on diet and/or maternal SES. For example, children exposed to high levels of DHA in utero may be strongly buffered against the negative effects of a stressful, low-income environment; whereas, children born into a more privileged environment and fed nutrient-rich foods may not benefit as much from prenatal DHA exposure. We therefore sought to succinctly characterize the diets and maternal SES of this cohort of US toddlers and preschool children using an unbiased, empiric approach so that we may later test for interactions with prenatal DHA supplementation. Our goals were to 1) efficiently summarize dietary intake into dietary patterns, 2) generate an SES score for each subject, and 3) describe how they are related in this unique cohort.

## Methods

### Subjects

Children studied were a subset of the Kansas University Docosahexaenoic Acid (DHA) Outcomes Study (KUDOS; NCT00266825) cohort [17]. Both the research protocol and informed consent adhered to the Declaration of Helsinki and ethical approval was obtained from the University of Kansas Institutional Review Board (HSC #11406).

### Data Collection

A registered dietitian assessed dietary intake on yearly and half-yearly anniversaries of the child’s birth from 2-4.5 years by 24-h recall using a standardized multiple-pass procedure [21]. Recalls were entered into Nutrition Data System for Research (NDSR) (versions 2006-2014, University of Minnesota) and were checked for accuracy and reliability by a second registered dietitian. Differences between coders were mutually reconciled. Children with at least one reliable dietary assessment were included in this analysis. There were 974 reliable dietary recalls among the 190 subjects, making the average number of dietary recalls 5.1 per participant. All six dietary recalls were available and reliable for 105 (55%) participants; five recalls for 42 (22%) participants; four recalls for 20 (11%) participants; three recalls for 11 (6%) participants; two recalls for 9 (5%) participants; and a single recall for 3 (2%) participants.

Baseline characteristics of the children’s mothers were obtained from the medical record and from interviews conducted at enrollment. Gestational weight gain and maternal body mass index (BMI, kg/m^2^) at enrollment were obtained from medical records. Other maternal characteristics, including smoking history, years of education, race and ethnicity were self-reported. Income was the median income by zip code at time of enrollment into the parent study. Birthweight and infant feeding history including breast and formula feeding were collected prospectively as part of the parent trial.

### Statistical Approach

The nutrient analysis software NDSR provided intake information on 168 food and beverage categories that the authors grouped into 34 categories in the same manner as Wosje et. al [22]. before further consolidating into 24 non-overlapping food and beverage groups by two registered dietitians (EK and JT). For example, the “added sugar” food category included: (a) sugar, (b) syrup, honey, jam, jelly, and preserves, (c) chocolate candy, (d) non-chocolate candy, (e) regular sweet sauces, (f) reduced fat or reduced calorie sweet sauces, and (g) frosting and glaze. As another example, the consolidated “sweet beverage” category included (a) sweetened soft drink, (b) sweetened fruit drink, (c) citrus juice, (d) non-citrus fruit juice, (e) sweetened tea, (f) sweetened flavored milk powder with milk, (g) sweetened flavored milk powder without milk, (g) sweetened coffee, (h) sweetened coffee substitutes, and (i) sweetened water. The full consolidation scheme is available in Additional file 1: Table S1. Spearman’s rank-correlation coefficient assessed whether intake of each food and beverage group increased or decreased over time.

The average daily intake of each food/beverage group during the 2.5 years period was calculated for each child after adjusting for the effect of age via residulization, that is subtracting the difference between mean intake at a specific time point and the grand mean intake for each of the 23,376 data points related to diet. This resulted in a single, mean estimated daily intake of each food and beverage group for each participant. Ward’s hierarchical cluster analysis [23] of the matrix of standardized (z-score) dietary data, 190 rows by 24 columns, suggested two mutually exclusive groups based on dendrogram inspection (Additional file 2: Figure S1). Children were characterized as falling into one of the two clusters which were named “Prudent” and “Western” diets, because they reflected similar dietary patterns found in other pediatric samples [22, 24–29].

An SES score was generated for each participant from maternal age, years of education, race/ethnicity (non-Hispanic White, non-Hispanic Black, Hispanic White or Other) and median income of maternal zip code using unrotated factor analysis, also known as principal components analysis [30], with the following equation:

$$ \mathrm{S}\mathrm{E}\mathrm{S} = 0.691*\mathrm{Age} + 0.826*\mathrm{Education} + 0.798*\mathrm{Income}\ \hbox{--}\ 0.71*\mathrm{Race} $$

where “Age” is the standardized maternal age at enrollment in years, “Education” is the standardized years of maternal education at enrollment, “Income” is the standardized logarithmic median household income of the mother’s zip code at enrollment, and “Race” is zero for mothers who self-identified as “non-Hispanic White” and “non-Hispanic Other;” and one for women who self-identified as “non-Hispanic Black” and “Hispanic White.” Maternal variables were selected based on prior research of important components of maternal SES [15] and a multivariate SES index in women with breast cancer [31]. One factor was retained based on Scree plot inspection (Additional file 3: Figure S2). Raw scores were standardized by converting them from eigenvalues to z-scores and then added to a constant to make all final SES scores positive with an integer mean.

The bivariate association between dietary pattern and categorical characteristics, that is maternal race/ethnicity and child sex, was assessed by Pearson’s chi-square test. Then, SES score was added as a covariate to assess the independent association between dietary pattern and each categorical characteristic using logistic regression. An interaction term, (dietary pattern)*(SES-μ_SES_), tested whether the association between dietary pattern and categorical characteristic differed by SES score.

For continuous variables, e.g., maternal height and birthweight, the bivariate association with dietary pattern was first assessed by the Mann-Whitney *U* test. Then SES score was added as a covariate to assess the independent association between dietary pattern and each continuous characteristic using linear regression. For the latter analyses, the following variables were log-transformed to normalize the distribution, as assessed by the Shapiro-Wilk W statistic: maternal zip code median income, maternal weight at enrollment, maternal BMI at enrollment, days of formula feeding, and days of breastfeeding. An interaction term, (dietary pattern)*(SES-μ_SES_), tested whether the association between dietary pattern and continuous characteristic differed by SES score. The Levene test assessed the difference in variance of maternal red blood cell (RBC) DHA at baseline and at delivery (following prenatal supplementation).

All statistical analyses were performed by BHH using JMP 12.0 (SAS Institute). The type I error rate was set at 5% (*P* ≤ 0.05 was considered significant) without adjustment for multiple comparisons. Instead, all P-values are presented.

## Results

Table 1 describes the 190 mother-child dyads. Only three participants reported “Other” race/ethnicity and they described themselves as “Pacific Islander or Native Hawaiian,” “Asian,” and “American Indian or Native Alaskan.” There was higher variance in maternal RBC DHA at delivery than at baseline (*p* < 0.0001).

**Table 1:** Mother-Child Characteristics

		N	% of total
Group	Placebo	91	48%
	DHA	99	52%
Race/Ethnicity	White	119	63%
	Black	56	29%
	Hispanic	12	6%
	Other	3	2%
Maternal Smoking	Never	103	54%
	Ever	87	46%
Breastfeeding^a^	None	38	20%
	Some	151	80%
Offspring Sex	Female	95	50%
	Male	95	50%
		Range	Mean ± SD
	Maternal age (yr)	16.1 to 36.0	26.1 ± 4.8
	Maternal education (yr)	9 to 20	14.6 ± 2.5
	Zip code income ($1 k)	18 to 154	48 ± 20
	Maternal height (cm)	146 to 182	164 ± 6.7
	Maternal weight (kg)	45.4 to 110.2	72.7 ± 13.6
	BMI at enrollment	16.5 to 42.6	27.3 ± 5.2
	Gestational weight gain (kg)	-2.7 to 25.9	12.8 ± 5.9
	Birthweight (g)	1290 to 4704	3348 ± 504
	Formula fed^a^ (days)	0 to 999	271 ± 158
	Breast fed^b^ (days)	0 to 1236	203 ± 244
	RBC DHA at enrollment^a^ (%)	1.73 to 8.60	4.36 ± 1.20
	RBC DHA at delivery^c^ (%)	2.50 to 12.26	6.15 ± 2.23
	RBC DHA change^d^ (%)	-5.29 to 7.32	1.79 ± 2.29
	Adherence (% of capsules consumed)	16 to 100	81.4 ± 19.1

^a^Missing data for *n* = 1

^b^Missing data for *n* = 2

^c^Missing data for *n* = 5

^d^Missing data for *n* = 6

The SES factor explained 58% of the variance in its four composite variables: maternal race, years of formal education, age, and income by zip code. Individual SES scores ranged from 0.12 to 4.02 with a mean ± SD of 2.00 ± 1.00.

The associations among SES and sample characteristics are exhibited in Table 2. The average non-Hispanic White mother’s SES score was 1.52 (95% confidence interval (CI): 1.29 to 1.74) higher than the average Black mother’s and 1.35 (95% CI: 0.93 to 1.78) higher than Hispanic White mother’s scores. Women who had never smoked tobacco had higher SES compared to those who had smoked (mean difference 0.36, 95% CI: 0.08 to 0.65). Women who breastfed had a higher SES score compared to those who fed their infant only formula (mean difference 0.73, 95% CI: 0.43 to 1.02). Maternal SES was positively associated with maternal height, gestational weight gain, birthweight, duration of breastfeeding, maternal RBC DHA at enrollment and delivery, change in DHA, and adherence and negatively associated with maternal weight, maternal BMI, and duration of formula feeding (all *p* < 0.05). Overweight and obese mothers (BMI ≥25) had a lower SES score (mean difference 0.48, 95% CI: 0.19 to 0.78).

**Table 2:** Associations among Maternal Socioeconomic Status Score and Maternal and Child Characteristics

		Mean ± SD	*P*-value^a^
Group	Placebo	1.92 ± 1.04	0.30
	DHA	2.08 ± 0.97	
Race/ethnicity	White	2.54 ± 0.75	<0.0001
	Black	1.02 ± 0.56	
	Hispanic	1.18 ± 0.92	
	Other	2.43 ± 0.53	
Maternal smoking	Never	2.17 ± 1.01	0.017
	Yes	1.81 ± 0.96	
Breast feeding	None	1.42 ± 0.76	<0.0001
	Some	2.14 ± 1.01	
Offspring sex	Female	1.98 ± 0.97	0.74
	Male	2.02 ± 1.05	
		Correlation^b^	*P*-value^b^
	Maternal age	0.69	<0.0001
	Maternal education (yr)	0.83	<0.0001
	Zip code income ($1 k)	0.80	<0.0001
	Maternal height (cm)	0.18	0.0135
	Maternal weight (kg)	-0.22	0.0023
	BMI at enrollment	-0.29	<0.0001
	Gestational weight gain (kg)	0.16	0.029
	Birthweight (g)	0.17	0.0227
	Formula fed(days)	-0.36	<0.0001
	Breast fed (days)	0.47	<0.0001
	RBC DHA at enrollment (%)	0.17	0.017
	RBC DHA at delivery (%)	0.32	<0.0001
	RBC DHA change (%)	0.22	0.0024
	Adherence (% of capsules consumed)	0.26	0.0003

^a^The *P*-value was calculated by the Mann-Whitney *U* test

^b^Spearman’s correlation and corresponding *P*-value

**Fig. 1:**
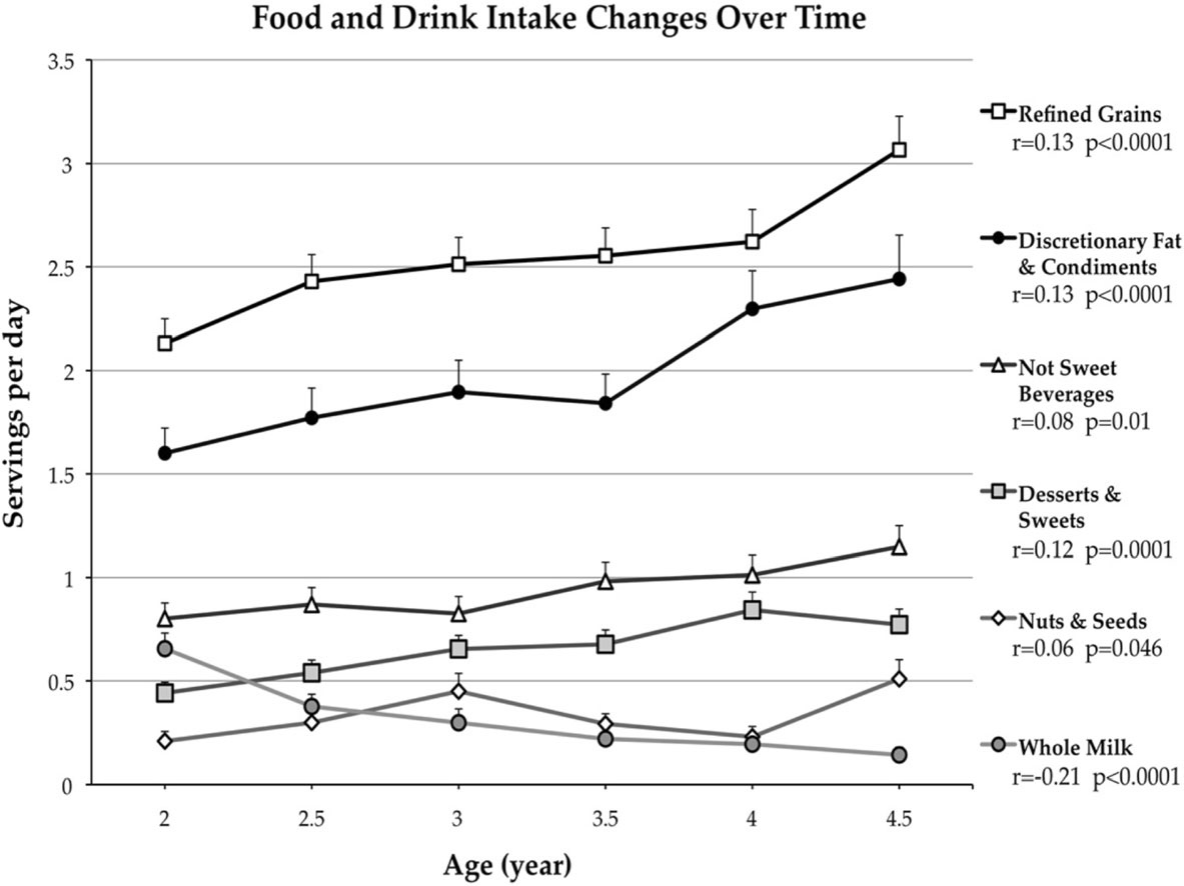
Food Groups and Beverage Categories Intake Changes from Age 2 to 4.5 Years. Mean daily intake of the 6 of 24 food groups that increased or decreased significantly over time during early childhood. Error bars are the standard error. Spearman’s correlations and corresponding p-values are shown

In decreasing order, the six most consumed food and beverage categories were 1) refined grains, 2) discretionary fat and condiments, 3) sweet beverages, 4) yogurt and non-whole milk, 5) artificially sweetened and unsweetened beverages (not sweet beverages), and 6) fruit. In servings, the mean ± SD daily consumption of total grains was 3.6 ± 1.1, total meat 2.0 ± 1.3, total dairy 1.7 ± 1.1, total fruit 0.8 ± 0.7, and total vegetables plus legumes 0.8 ± 0.7. Intake of only 6 of the 24 food and beverage categories changed significantly over the 2.5 years period as depicted in Fig. 1 (all *p* < 0.05). From 2 to 4.5 years of age, the average daily intake of refined grains increased by 44%, discretionary fat and condiments by 53%, not sweetened beverages by 43%, desserts and sweets by 75%, and nuts and seeds by 145% while mean daily whole milk intake decreased by 78%.

**Fig. 2:**
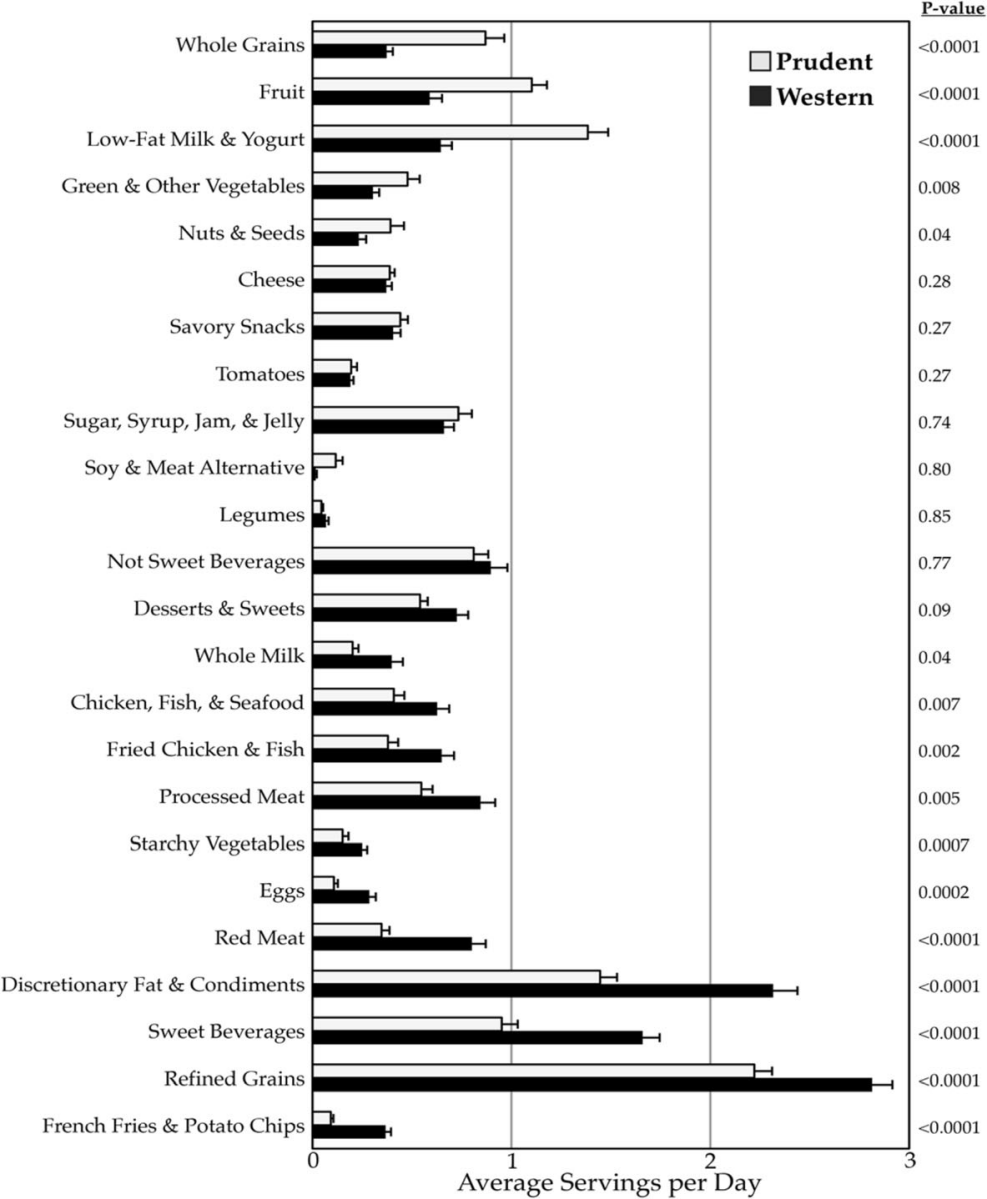
Average Daily Food and Beverage Intake of the Dietary Pattern Clusters. Error bars are standard error. P-values were calculated by the Mann-Whitney *U* test

Cluster analysis identified two major dietary patterns. Eighty-five children (45%) had a Prudent diet and 105 children (55%) had a Western diet. As illustrated in Fig. 2, which includes all 24 final food and beverage groupings, the Prudent diet (versus the Western diet) was principally characterized by *greater* consumption of whole grains, fruit, and yogurt and low-fat milk; and *lower* consumption of red meat, discretionary fat and condiments, sweet beverages, refined grains, and French fries and potato chips (all *p* < 0.0001). Smaller magnitude differences included children with a Prudent diet consuming *more* green and non-starchy vegetables and nuts and seeds, and *less* eggs, starchy vegetables, processed meats, fried and not fried chicken and seafood, and whole milk (all *p* < 0.05).

Table 3 presents the associations between the child dietary patterns and characteristics of mothers and children. Compared to the mothers of children with a Western dietary pattern, mothers of children with a Prudent diet had a mean SES score that was one standard deviation higher (difference 1.05, 95% CI: 0.80 to 1.30). Compared to children with a White mother, children with a Black mother were twice as likely to have a Western dietary pattern (relative risk 2.1, 95% CI: 1.6 to 2.7). Mothers of children with a Prudent diet had higher RBC DHA at baseline; relative difference 12 % (95% CI: 3.6 to 20%); however, this association lost strength with adjustment for SES. After controlling for maternal SES, the mothers of children with a Prudent diet, on average were 1.9% taller (*p* = 0.03), had a 9.8% lower BMI (*p* = 0.04), breastfed 110% longer (*p* = 0.002), were 24% more likely to have breastfed at all (*p* = 0.01), and were more adherent (*p* = 0.004), i.e., they took 8.8% (95% CI: 2.8 to 15%) more of their capsules. There was no evidence of an interaction between SES and dietary pattern for any maternal characteristic (all *p* > 0.05), except for adherence. The women with the lowest adherence were of low SES and reported a Prudent diet for their offspring (p_interaction_ = 0.0007).

**Table 3:** Associations of Dietary Patterns with Maternal and Child Characteristics

		Prudent Pattern (*n* = 85)	Western Pattern (*n* = 105)		
		n (column%)	n (column%)	*P*-value^a^	*P*-value^b^
Group	Placebo	41 (48%)	50 (48%)	0.94	0.46
	DHA	44 (52%)	55 (52%)		
Race/Ethnicity	White	71 (84%)	48 (46%)	<0.0001	0.24
	Black	9 (11%)	47 (45%)		
	Hispanic	5 (6%)	7 (7%)		
	Other	0 (0%)	3 (3%)		
Maternal Smoking	Never	48 (56%)	55 (52%)	0.57	0.38
	Ever	37 (44%)	50 (48%)		
Breast Feeding	None	6 (7%)	32 (31%)	<0.0001	0.01
	Some	79 (93%)	72 (69%)		
Offspring Sex	Female	47 (55%)	48 (46%)	0.19	0.089
	Male	38 (45%)	57 (54%)		
		Mean ± SD	Mean ± SD		
	Maternal age	27.7 ± 4.1	24.8 ± 4.9	<0.0001	0.18
	Maternal education (yr)	16.0 ± 2.3	13.5 ± 2.1	<0.0001	0.068
	Zip code income ($1 k)	57 ± 22	41 ± 15	<0.0001	0.85
	Maternal height (cm)	165.2 ± 6.5	162.1 ± 6.5	0.0024	0.028
	Maternal weight (kg)	70.1 ± 12.2	74.8 ± 14.3	0.015	0.28
	BMI at enrollment	25.7 ± 4.3	28.5 ± 5.5	0.0002	0.038
	Gestational weight gain (kg)	13.7 ± 5.7	12.1 ± 6.0	0.12	0.51
	Birthweight (g)	3386 ± 550	3318 ± 464	0.15	0.94
	Formula feeding duration (days)	225 ± 171	308 ± 137	<0.0001	0.13
	Breast feeding duration (days)	286 ± 251	136 ± 216	<0.0001	0.0021
	RBC DHA at enrollment (%)	4.61 ± 1.24	4.14 ± 1.12	0.0043	0.076
	RBC DHA at delivery (%)	6.44 ± 2.46	5.92 ± 2.01	0.22	0.37
	RBC DHA Change (%)	1.80 ± 2.35	1.79 ± 2.26	0.91	0.076
	Adherence (% of capsules consumed)	81.4 ± 21.5	81.4 ± 17.1	0.41	0.0041
	SES Factor	2.58 ± 0.84	1.53 ± 0.87	<0.0001	-------

^a^The *P*-value was calculated by Pearson’s Chi-Square or the Mann-Whitney *U* test

^b^The *P*-value represents the association between dietary pattern and the maternal or child characteristic when controlling for SES

**Fig. 3:**
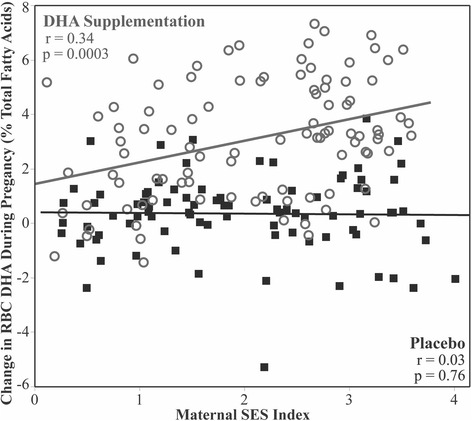
Red blood cell (RBC) Change and SES Score. The change in RBC DHA is related to randomization (placebo or DHA supplementation) and SES score. The effect of supplementation on maternal RBC DHA differed by SES (p_interaction_ = 0.002); high SES was associated with a larger increase in DHA among those who received DHA (*r* = 0.34, *p* = 0.0007), but not among those who received placebo (*p* = 0.76)

Supplementation affected maternal RBC DHA composition (*p* < 0.0001); mothers randomized to the control group increased their DHA 0.35% (95% CI: -0.04 to 0.74%) while mothers who received active treatment increased their DHA 3.09% (95% CI: 2.72 to 3.46%). The effect of supplementation on maternal RBC DHA differed by SES (p_interaction_ = 0.002); high SES was associated with a larger increase in DHA among those who received DHA (*r* = 0.34, *p* = 0.0007), but not among those who received placebo (*p* = 0.**76**) (Fig. 3). Interestingly, the interaction between SES, supplementation, and change in DHA persisted even after controlling for adherence (p_interaction_ = 0.002). In fact, adherence was not significantly associated with change in maternal RBC DHA among women who received active treatment (*r* = 0.13, *p* = 0.20).

## Discussion

We have characterized the dietary habits of a diverse and unique cohort of young children in the US and reported how a child’s major dietary pattern relates to maternal SES and other maternal characteristics. We found that daily food and drink intake is relatively stable across the ages of 2 to 4.5 years, in general agreement with data from longitudinal studies conducted in Europe [10, 14]. However, it was concerning that the young children mostly added energy-dense foods of low nutritional value (refined grains, discretionary fat and condiments, and desserts and sweets) as they grew older.

We chose to average a child’s intake over time in order to maximize our sample size (i.e., include children with missing data) while attempting to minimize the effect of age via residualization. Using average intake over the 2.5 years period, we categorized the pattern of food and drink consumption in early childhood as either Prudent or Western. A Prudent diet represented relatively greater consumption of whole grains, fruit, yogurt and low-fat milk, green and non-starchy vegetables, and nuts and seeds. In contrast, children with a Western diet ate more red meat, discretionary fat and condiments, sweet beverages, refined grains, French fries and potato chips, eggs, baked or boiled starchy vegetables, processed meats, fried chicken and seafood, not-fried chicken & seafood, and whole milk.

We used cluster analysis to create a single, categorical variable from the large amount of information describing the diets of children age 2 to 4.5 years. We chose to avoid dietary factor analysis, because (a) categorical variables are easier to comprehend compared to continuous variables, (b) it can be difficult to justify how many factors are retained (c) we wanted to minimize the number of newly constructed variables, and (d) factor scores are usually categorized into quantiles, a practice that has been critiqued [32]. Other groups of investigators have used reduced rank regression (RRR) as a multivariate technique to identify dietary patterns that explain maximum variance of outcomes [22, 28], however, defining dietary pattern by RRR could limit future evaluations. The children in this cohort are being extensively phenotyped (e.g., cognitive testing, blood pressure, body composition, brain structure/function and growth) as they age to 9 years. The empirically defined dietary patterns herein are not biased toward prediction of any outcome; however, they are not optimized for any outcome either.

We found that several important maternal features differed by offspring dietary pattern. Mothers of children with a Western dietary pattern had a lower SES, breastfed less, and had a higher BMI at enrollment; observations similar to what was found for the “Junk” dietary pattern from the Avon Longitudinal Study of Parents and Children (ALSPAC) cohort [33]. Our findings are also congruent with the direct association between parental education and more favorable nutrient intake among older female children in the US [34]. We found evidence that a child’s diet was related to his or her mother’s dietary behavior, because higher maternal DHA at baseline was associated with the offspring consuming a Prudent diet. Interestingly, maternal height was positively associated with a child adhering to a Prudent dietary pattern even after adjusting for SES. Children with a Prudent diet consumed more low-fat milk and yogurt; this observation is consistent with the hypothesis that milk protein intake is associated with greater height in children [35, 36].

Mothers who were non-Hispanic White, older, more educated, and lived in a higher income area had elevated SES scores, while mothers who were Black or Hispanic, younger, less educated, and lived in an economically disadvantaged neighborhood had lower SES scores. The collinearity among these variables is consistent with other US samples showing that education is positively correlated with age at first birth [37] and income [38]. Black and Hispanic families are about twice as likely to live in poverty compared to Asian and non-Hispanic White households [38]. Individuals with higher income tend to be taller [39], which is consistent with the positive correlation between SES and maternal height that we observed. We also found that SES was inversely correlated with maternal weight and BMI at enrollment, which is compatible with known associations between weight status, race, and education [40]. The more weight a mother gains during pregnancy, the greater the offspring’s weight at birth [41]. A positive correlation between SES and gestational weight gain and birthweight parallels the association between poverty and likelihood of intrauterine growth restriction [42]. We replicated the positive correlation between breastfeeding and SES [43]. The congruency between the SES associations in this report and previous research corroborates the validity of the SES index we constructed.

There was a large range in effect of supplementation on maternal RBC DHA concentration. DHA levels were stable for those randomized to control, regardless of maternal SES or adherence; however, for those mothers randomized to DHA supplementation, maternal SES was a stronger predictor of DHA increase than pill count. There was an interesting interaction between maternal SES and child dietary pattern for adherence. Low SES mothers that reported a Prudent diet for their child had the worst adherence by pill count; this may indicate a lack of candor with regard to dietary recall. The observation is limited to a small number of participants and could be spurious; nevertheless, it will be interesting to see if this subset of children exhibits a developmental trajectory more similar to those with a Western dietary pattern.

Our study has several limitations. First, the dietary pattern analysis is susceptible to (a) inaccurate recalls from caregivers reporting healthier dietary habits than reality, (b) a sample biased by loss to follow-up, (c) the constraints of the NDSR’s categories of food and beverages, and (d) our food/beverage group consolidation scheme. Second, for the sake of parsimony, cluster analysis admittedly discards a large amount of the variance. Third, SES is a multidimensional construct that includes measurements not included here, e.g., accumulated economic assets, parity, occupation, marital status, and paternal income [15]. Fourth, we did not directly measure family income, but instead used a residential proxy. Fifth, we incorporated age and race/ethnicity into the SES index, because they are likely mediators of SES effects on maternal-fetal health; however, this assumes that the associations among education level, income, and age are stable across racial/ethnic categories, an assumption that may not be true [15]. Lastly, the major tradeoff of extracting the maximum SES signal from available variables is that this obviates the potential to assess independent effects of the covariates that comprise SES in future analyses.

## Conclusions

We observed a consistent number of servings per day consumed for most food and beverage categories across the period of 2 to 4.5 years of age, defined two major and mutually exclusive dietary patterns in a unique sample of US preschool children and a single, continuous axis of maternal SES, and identified significant relationships between dietary pattern and maternal variables that were present even after adjusting for maternal SES. We look forward to exploring how a young child’s diet, maternal SES, and prenatal supplementation of DHA interact to influence child growth and development.

## Additional files

Additional file 1: Table S1. Original Food and Beverage Variables. Contains all food and beverage variables and their consolidation into one of 24 good groups. (DOCX 133 kb)
Additional file 2: Figure S1. Dendrogram illustrating two mutually exclusive dietary patterns based on evaluation of all 190 children’s mean estimated daily intake of each of 24 food and beverage groups. In the dendrogram, each row represents one participant. The x-axis represents the average difference between groups (or individual subjects) at a given branch point. Branch points toward the left indicate that the joining groups are more similar to one another. (DOCX 506 kb)
Additional file 3: Figure S2. Scree plot of unrotated factor analysis of maternal SES variables. The four raw variables (maternal age, years of maternal education, race/ethnicity, and median income of maternal zipcode) were analyzed by unrotated factor analysis. Each factor is worth one eigenvalue. The Scree plot represents the portion of the variance (4 eigenvalues) explained by each factor. Only one factor is retained, because factors 2, 3, and 4 each explain an equally small amount of the total variance in the original variables. (DOCX 266 kb)

## Abbreviations

DHA: Docosahexaenoic acid
NDSR: Nutrition Data System for Research
RBC: Red blood cell
SES: Socioeconomic status

## References

[1] Handel MN, Heitmann BL. Nutrient and food intakes in early life and risk of childhood fractures: a systematic review and meta-analysis. Am J Clin Nutr. 2015;102(5):1182-95. 10.3945/ajcn.115.108456 26447151

[2] Smith AD, Emmett PM, Newby PK, Northstone K. Dietary patterns and changes in body composition in children between 9 and 11 years. Food Nutrition Res. 2014;58:1-8. 10.3402/fnr.v58.22769 PMC4090365 25018688

[3] Haapala EA, Eloranta AM, Venalainen T, Schwab U, Lindi V, Lakka TA (2015). Associations of diet quality with cognition in children - the Physical Activity and Nutrition in Children Study. Br J Nutr.

[4] Golley RK, Smithers LG, Mittinty MN, Emmett P, Northstone K, Lynch JW (2013). Diet quality of U.K. infants is associated with dietary, adiposity, cardiovascular, and cognitive outcomes measured at 7-8 years of age. J Nutr.

[5] Brazionis L, Golley RK, Mittinty MN (2013). Diet spanning infancy and toddlerhood is associated with child blood pressure at age 7.5 y. Am J Clin Nutr.

[6] Pala V, Lissner L, Hebestreit A (2013). Dietary patterns and longitudinal change in body mass in European children: a follow-up study on the IDEFICS multicenter cohort. Eur J Clin Nutr.

[7] van Zon SKR, Bültmann U, de Leon CF M, Reijneveld SA (2015). Absolute and relative socioeconomic health inequalities across age groups. PLoS One.

[8] Martin MA, Van Hook JL, Quiros S (2015). Is socioeconomic incorporation associated with a healthier diet? Dietary patterns among Mexican-origin children in the United States. Soc Sci Med.

[9] Perez-Rodrigo C, Gil A, Gonzalez-Gross M, et al. Clustering of Dietary Patterns, Lifestyles, and Overweight among Spanish Children and Adolescents in the ANIBES Study. Nutrients. 2015;8(1):1-17. 10.3390/nu8010011 PMC4728625 26729155

[10] Emmett PM, Jones LR, Northstone K (2015). Dietary patterns in the avon longitudinal study of parents and children. Nutr Rev.

[11] Bortolini GA, Vitolo MR, Gubert MB, Santos LM (2015). Social inequalities influence the quality and diversity of diet in Brazilian children 6 to 36 months of age. Cad Saude Publica.

[12] Leventakou V, Sarri K, Georgiou V (2016). Early life determinants of dietary patterns in preschool children: Rhea mother-child cohort, Crete, Greece. Eur J Clin Nutr.

[13] Fernandez-Alvira JM, Bammann K, Pala V (2014). Country-specific dietary patterns and associations with socioeconomic status in European children: the IDEFICS study. Eur J Clin Nutr.

[14] Fernandez-Alvira JM, Bornhorst C, Bammann K (2015). Prospective associations between socio-economic status and dietary patterns in European children: the Identification and Prevention of Dietary- and Lifestyle-induced Health Effects in Children and Infants (IDEFICS) Study. Br J Nutr.

[15] Braveman P, Cubbin C, Marchi K, Egerter S, Chavez G (2001). Measuring socioeconomic status/position in studies of racial/ethnic disparities: maternal and infant health. Public Health Rep.

[16] Huang RC, Prescott SL, Godfrey KM, Davis EA (2015). Assessment of cardiometabolic risk in children in population studies: underpinning developmental origins of health and disease mother-offspring cohort studies. J Nutr Sci.

[17] Carlson SE, Colombo J, Gajewski BJ (2013). DHA supplementation and pregnancy outcomes. Am J Clin Nutr.

[18] Madden J, Williams CM, Calder PC (2011). The impact of common gene variants on the response of biomarkers of cardiovascular disease (CVD) risk to increased fish oil fatty acids intakes. Annu Rev Nutr.

[19] Wells EM, Herbstman JB, Lin YH (2016). Cord blood methylmercury and fetal growth outcomes in baltimore newborns: potential confounding and effect modification by omega-3 fatty acids, selenium, and sex. Environ Health Perspect.

[20] Bigornia SJ, Harris WS, Falcon LM, Ordovas JM, Lai CQ, Tucker KL (2016). The omega-3 index is inversely associated with depressive symptoms among individuals with elevated oxidative stress biomarkers. J Nutr.

[21] Johnson RK, Driscoll P, Goran MI (1996). Comparison of multiple-pass 24-hour recall estimates of energy intake with total energy expenditure determined by the doubly labeled water method in young children. J Am Diet Assoc.

[22] Wosje KS, Khoury PR, Claytor RP (2010). Dietary patterns associated with fat and bone mass in young children. Am J Clin Nutr.

[23] Ward JH (1963). Hierarchical grouping to optimize an objective function. J Am Stat Assoc.

[24] Hoffmann K, Schulze MB, Schienkiewitz A, Nöthlings U, Boeing H (2004). Application of a new statistical method to derive dietary patterns in nutritional epidemiology. Am J Epidemiol.

[25] Ambrosini GL, Emmett PM, Northstone K, Jebb SA (2014). Tracking a dietary pattern associated with increased adiposity in childhood and adolescence. Obesity.

[26] Shin KO, Oh S-Y, Park HS (2007). Empirically derived major dietary patterns and their associations with overweight in Korean preschool children. Br J Nutr.

[27] Shang X, Li Y, Liu A (2012). Dietary pattern and its association with the prevalence of obesity and related cardiometabolic risk factors among Chinese children. PLoS One.

[28] Johnson L, Mander AP, Jones LR, Emmett PM, Jebb SA (2008). Energy-dense, low-fiber, high-fat dietary pattern is associated with increased fatness in childhood. Am J Clin Nutr.

[29] Diethelm K, Gunther AL, Schulze MB, Standl M, Heinrich J, Buyken AE (2014). Prospective relevance of dietary patterns at the beginning and during the course of primary school to the development of body composition. Br J Nutr.

[30] Hotelling H (1933). Analysis of a complex of statistical variables into principal components. J Educ Psychol.

[31] Yost K, Perkins C, Cohen R, Morris C, Wright W. Socioeconomic status and breast cancer incidence in California for different race/ethnic groups. Cancer Causes Control. 2001;12(8):703-11. 10.1023/a:1011240019516 11562110

[32] Bennette C, Vickers A (2012). Against quantiles: categorization of continuous variables in epidemiologic research, and its discontents. BMC Med Res Methodol.

[33] Wiles NJ, Northstone K, Emmett P, Lewis G (2009). 'Junk food' diet and childhood behavioural problems: results from the ALSPAC cohort. Eur J Clin Nutr.

[34] Crawford PB, Obarzanek E, Schreiber GB (1995). The effects of race, household income, and parental education on nutrient intakes of 9-and 10-year-old girls NHLBI growth and health study. Ann Epidemiol.

[35] Berkey CS, Colditz GA, Rockett HR, Frazier AL, Willett WC (2009). Dairy consumption and female height growth: prospective cohort study. Cancer Epidemiol Biomarkers Prev.

[36] Wiley AS (2009). Consumption of milk, but not other dairy products, is associated with height among US preschool children in NHANES 1999–2002. Ann Hum Biol.

[37] Heck K, Schoendorf K, Ventura S, Kiely J (1997). Delayed childbearing by education level in the United States, 1969–1994. Matern Child Health J.

[38] DeNavas-Walt CBDP, Bureau USC (2015). Income and Poverty in the United States: 2014. Vol Current Population Reports.

[39] Meyer HE, Selmer R (1999). Income, educational level and body height. Ann Hum Biol.

[40] Paeratakul S, Lovejoy J, Ryan D, Bray G (2002). The relation of gender, race and socioeconomic status to obesity and obesity comorbidities in a sample of US adults. Int J Obesity Relat Metabol Disord.

[41] Frederick I, Williams M, Sales A, Martin D, Killien M (2008). Pre-pregnancy body mass index, gestational weight gain, and other maternal characteristics in relation to infant birth weight. Matern Child Health J.

[42] Kramer MS, Séguin L, Lydon J, Goulet L (2000). Socio-economic disparities in pregnancy outcome: why do the poor fare so poorly?. Paediatr Perinat Epidemiol.

[43] Heck KE, Braveman P, Cubbin C, Chávez GF, Kiely JL (2006). Socioeconomic status and breastfeeding initiation among California mothers. Public Health Rep.

