# Supplementary material for: Dietary patterns of early childhood and maternal socioeconomic status in a unique prospective sample from a randomized controlled trial of Prenatal DHA Supplementation
Source: BMC Pediatr. 2016 Nov 25;16:191. doi: 10.1186/s12887-016-0729-0 (PMC5123236; doi:10.1186/s12887-016-0729-0)
Supplement: Additional file 1: Table S1. — Original Food and Beverage Variables. Contains all food and beverage variables and their consolidation into one of 24 good groups. (DOCX 133 kb) [file 12887_2016_729_MOESM1_ESM.docx]

| **Final 24 Groups** | **Original Food and Beverage Variables** |
| --- | --- |
| Whole grains | Whole grain bread |
|  | Whole grain other bread (quick breads, corn muffins, tortillas) |
|  | Whole grain pasta |
|  | Unsweetened whole grain ready-to-eat cereal |
|  | Sweetened whole grain ready-to-eat cereal |
|  | Whole grain snack bars |
|  | Whole grain mix (brown rice, cracked wheat, oatmeal, corn meal, rye meal, whole wheat flour, etc.) |
| Refined grains | Refined grain bread |
|  | Mixed (whole + refined) grain bread |
|  | Refined grain other bread (quick breads, corn muffins, tortillas) |
|  | Mixed grain other bread (quick breads, corn muffins, tortillas) |
|  | Refined grain pasta |
|  | Mixed grain pasta |
|  | Unsweetened refined grain ready-to-eat cereal |
|  | Unsweetened mixed grain ready-to-eat cereal |
|  | Sweetened refined grain ready-to-eat cereal |
|  | Sweetened mixed grain ready-to-eat cereal |
|  | Refined grain snack bars |
|  | Mixed grain snack bars |
|  | Refined grain mix (corn meal, pearled barley, rye flour, white wheat flour, white rice, etc.) |
|  | Mixed grain mix (oat bran, rice bran, wheat germ, etc.) |
| Savory snacks | Whole grain crackers |
|  | Refined grain crackers |
|  | Mixed grain crackers |
|  | Whole grain snack chips (corn chips, rice cakes, etc.) |
|  | Refined grain snack chips (cheese puffs, pretzels, etc.) |
|  | Mixed grain snack chips (wheat nuts, etc.) |
|  | Popcorn |
|  | Flavored popcorn (caramel, cheese, etc.) |
| French fries & potato chips | Fried potatoes (French fries, hash browns, pan fried potatoes, fried potato skins, etc.) |
|  | Fried vegetables (breaded and fried broccoli, mushrooms, onion rings, etc. Excludes stir-fry or sauté) |
|  | Vegetable-based savory snack (potato chips, canned onion rings, etc.) |
| Starchy veggies | White potatoes (mashed potatoes, potato salad, scalloped potatoes, etc.) |
|  | Other starchy vegetables (corn, peas, peas, etc.) |
| Green and other vegetables | Dark green vegetables (broccoli, collards, romaine, spinach, etc.) |
|  | Deep yellow vegetables (carrots, winter squach, sweet potatoes, pumpkin, etc.) |
|  | Other vegetables (beets, cabbage, mung bean sprouts, summer squash, etc.) |
| Tomatoes | Tomatoes (raw, salsa, tomato sauce, tomato based spaghetti sauce, tomato puree, and tomato paste) |
| Legumes | Legumes (mature lima beans, refried beans, baked beans, pork and beans, black beans, etc.) |
| Fruit | Citrus fruit |
|  | Non-citrus fruit (includes fruit in cereal, excludes fruit in juice, candy, ice cream, granola bars, pie, cake, and other baked goods) |
| Nuts and Seeds | Nuts and seeds |
|  | Nut and seed butters |
| Red meat | Beef |
|  | Lean beef |
|  | Veal |
|  | Lean veal |
|  | Lamb |
|  | Lean lamb |
|  | Fresh pork |
|  | Lean fresh pork |
|  | Game |
|  | Organ meats |
| Processed meat | Cured pork |
|  | Lean cured pork |
|  | Cold cuts & sausage |
|  | Lean cold cuts & sausage |
| Chicken, fish, and seafood | Poultry (chicken, turkey, duck, quail, etc.) |
|  | Lean poultry |
|  | Fish (fresh & smoked) |
|  | Lean fish (fresh & smoked) |
|  | Shellfish |
| Fried chicken and fish | Fried chicken (commercial entrée and fast food) |
|  | Fried fish (commercial entrée and fast food) |
|  | Fried shellfish (commercial entrée and fast food) |
| Eggs | Eggs |
|  | Egg substitute |
| Soy and meat alternative | Meat alternatives (tofu, veggie burgers, etc.) |
|  | Non-dairy cream |
|  | Non-dairy yogurt |
|  | Non-dairy cheese |
|  | Non-dairy milk |
| Whole milk | Whole-fat milk |
|  | Whole-fat ready-to-drink flavored milk |
| Yogurt and non-whole mik | Reduced-fat milk |
|  | Low-/Fat-free milk |
|  | Reduced-fat ready-to-drink flavored milk |
|  | Low-/Fat-free ready-to-drink flavored milk |
|  | Sweetened whole-fat yogurt |
|  | Artificially sweetened whole-fat yogurt |
|  | Sweetened low-fat yogurt |
|  | Artificially sweetened low-fat yogurt |
|  | Sweetened non-fat yogurt |
|  | Artificially sweetened non-fat yogurt |
| Cheese | Full-fat cheese (includes natural and processed cheese, cottage cheese, cream cheese, etc.) |
|  | Reduced-fat cheese (includes natural and processed cheese, cottage cheese, cream cheese, etc.) |
|  | Low-/Non-fat cheese (includes natural and processed cheese, cottage cheese, cream cheese, etc.) |
| Desserts and sweets | Frozen dairy dessert (frozen yogurt, ice cream, ice cream treats, etc.) |
|  | Frozen non-dairy dessert |
|  | Pudding and other dairy dessert |
|  | Artificially sweetened pudding and other dairy dessert |
|  | Whole grain cake, cookies, pastries (muffins), Danish, doughnuts, cobblers, pies |
|  | Refined grain cake, cookies, pastries (muffins), Danish, doughnuts, cobblers, pies |
|  | Mixed grain cake, cookies, pastries (muffins), Danish, doughnuts, cobblers, pies |
| Added sugar | Sugar |
|  | Syrup, honey, jam, jelly, preserves |
|  | Sweet sauces |
|  | Reduced-fat/-calorie sweet sauces |
|  | Chocolate candy |
|  | Non-chocolate candy |
|  | Frosting or glaze |
| Discretionary fat and condiments | Whole-fat cream |
|  | Reduced-fat cream |
|  | Low-/non-fat cream |
|  | Margarine |
|  | Reduced fat margarine |
|  | Oil (canola, soy, sunflower, safflower, peanut, vegetable, etc.) |
|  | Shortening |
|  | Butter and other animal fats |
|  | Reduced fat butter and other animal fats |
|  | Salad dressing (includes mayonnaise) |
|  | Reduced fat salad dressing (includes mayonnaise) |
|  | Gravy |
|  | Low-/non-fat gravy |
|  | Sauces and condiments (alfredo sauce, cheese sauce, hollandaise sauce, tartar sauce, white sauce, etc.) |
|  | Reduced fat sauces and condiments (barbeque sauce, catsup, mustard, soy sauce, steak sauce, hot sauce, taco sauce, etc.) |
| Sweet beverages | Citrus juice |
|  | Not-citrus fruit juice |
|  | Sweetened fruit drink |
|  | Sweetened soft drink |
|  | Sweetened flavored milk powder with milk |
|  | Sweetened flavored milk powder without milk |
|  | Sweetened tea |
|  | Sweetened coffee |
|  | Sweetened coffee-substitutes |
|  | Sweetened water |
| Not sweet beverages | Artificially sweetened soft drink |
|  | Unsweetened soft drink |
|  | Artificially sweetened tea |
|  | Unsweetened tea |
|  | Artificially sweetened coffee |
|  | Unsweetened coffee |
|  | Artificially sweetened coffee substitutes |
|  | Unsweetened coffee substitutes |
|  | Artificially sweetened water |
|  | Vegetable juice |
