# Supplementary figures and images for: Dietary patterns of early childhood and maternal socioeconomic status in a unique prospective sample from a randomized controlled trial of Prenatal DHA Supplementation
Source: BMC Pediatr. 2016 Nov 25;16:191. doi: 10.1186/s12887-016-0729-0 (PMC5123236; doi:10.1186/s12887-016-0729-0)

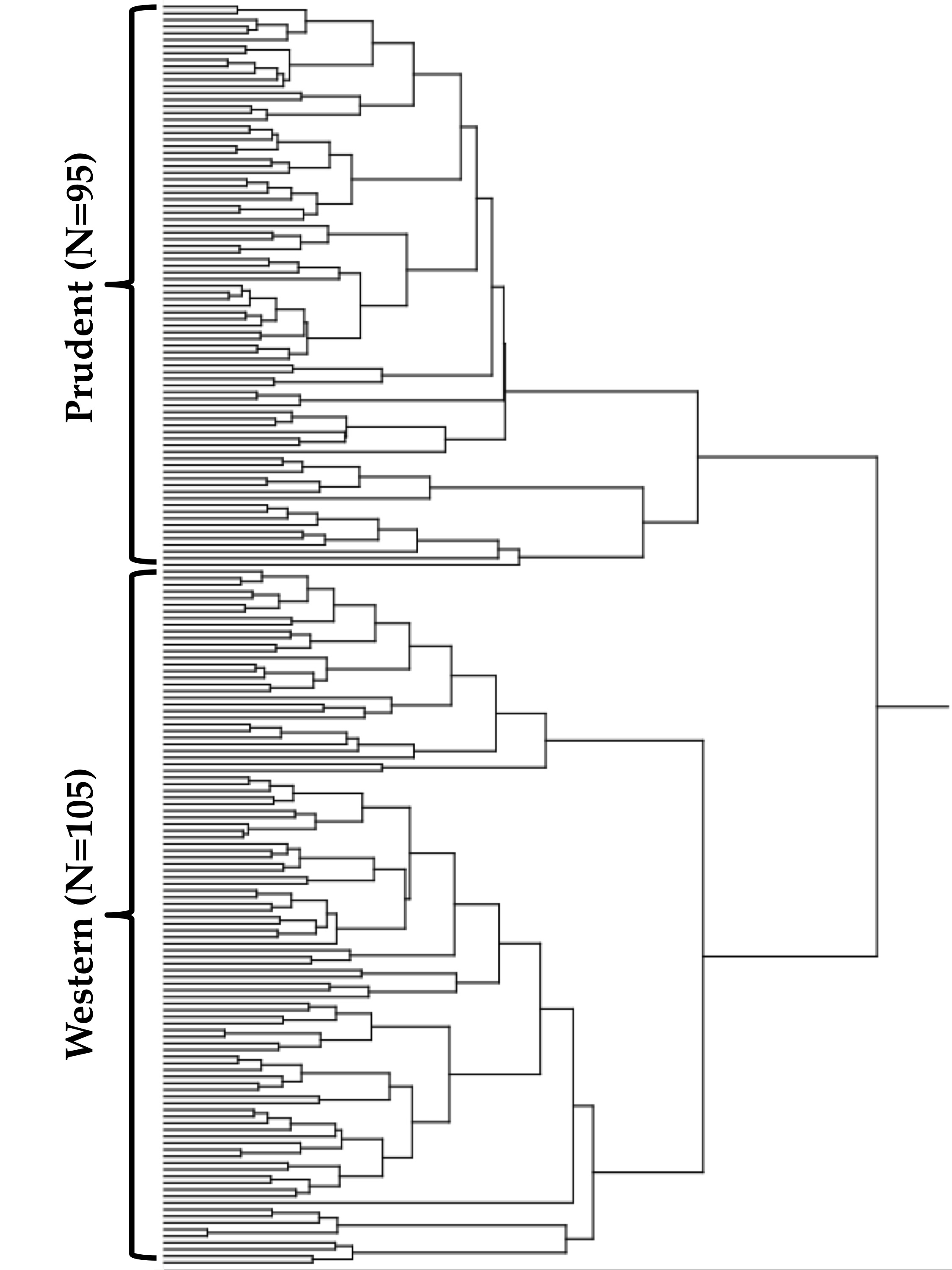

Supplement: Additional file 2: Figure S1. — Dendrogram illustrating two mutually exclusive dietary patterns based on evaluation of all 190 children’s mean estimated daily intake of each of 24 food and beverage groups. In the dendrogram, each row represents one participant. The x-axis represents the average difference between groups (or individual subjects) at a given branch point. Branch points toward the left indicate that the joining groups are more similar to one another. (DOCX 506 kb) [file 12887_2016_729_MOESM2_ESM.docx]

S
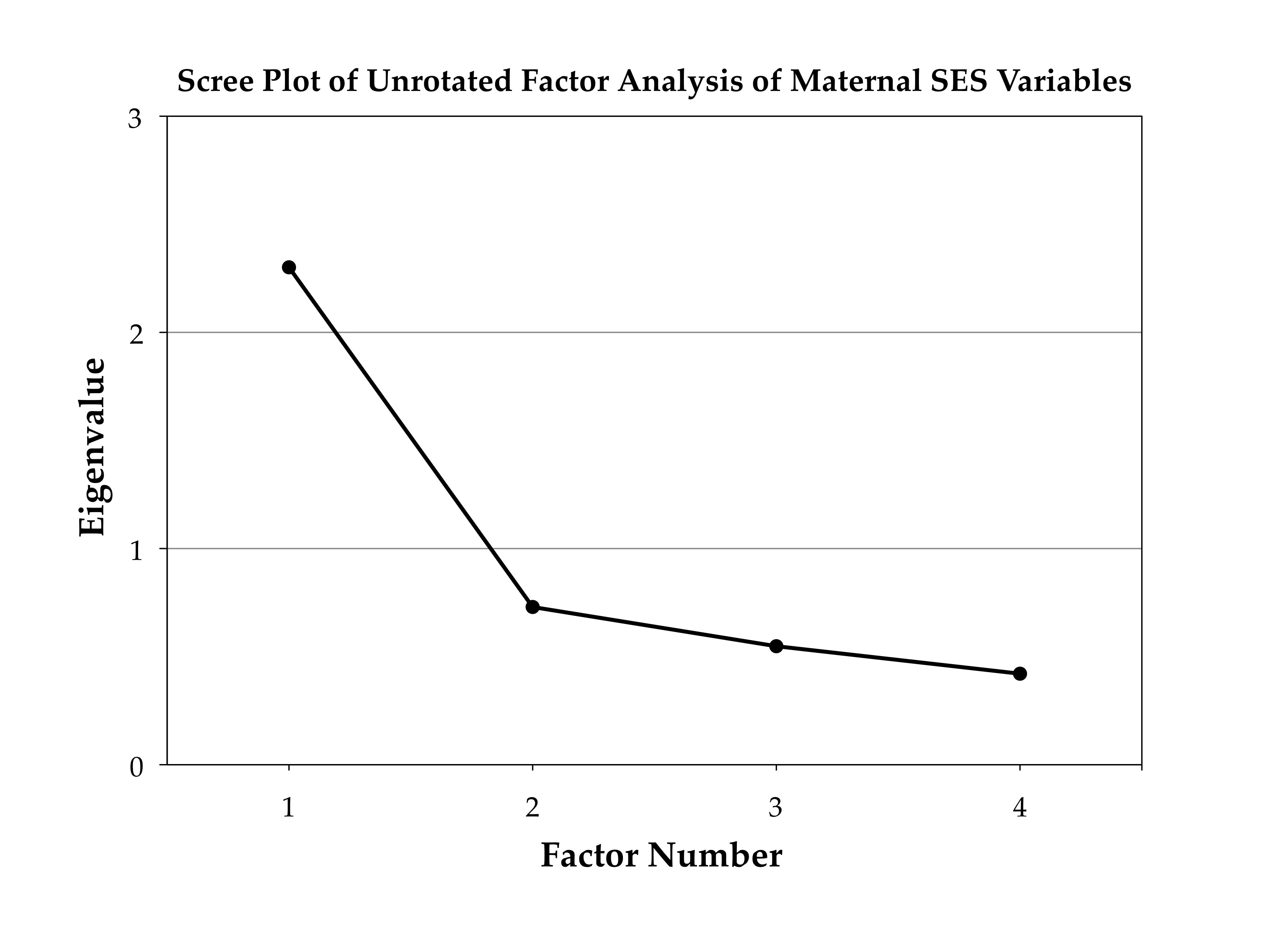


Supplemental Figure 2

Supplement: Additional file 3: Figure S2. — Scree plot of unrotated factor analysis of maternal SES variables. The four raw variables (maternal age, years of maternal education, race/ethnicity, and median income of maternal zipcode) were analyzed by unrotated factor analysis. Each factor is worth one eigenvalue. The Scree plot represents the portion of the variance (4 eigenvalues) explained by each factor. Only one factor is retained, because factors 2, 3, and 4 each explain an equally small amount of the total variance in the original variables. (DOCX 266 kb) [file 12887_2016_729_MOESM3_ESM.docx]
